# Acute Myeloid Leukemia-Targeted Toxin Activates Both Apoptotic and Necroptotic Death Mechanisms

**DOI:** 10.1371/journal.pone.0003909

**Published:** 2008-12-11

**Authors:** Henrick Horita, Arthur E. Frankel, Andrew Thorburn

**Affiliations:** 1 Department of Pharmacology, University of Colorado Denver School of Medicine, Aurora, Colorado, United States of America; 2 Scott & White Cancer Research Institute, Temple, Texas, United States of America; City of Hope Medical Center, United States of America

## Abstract

**Background:**

Acute myelogenous leukemia (AML) is the second most common leukemia with approximately 13,410 new cases and 8,990 deaths annually in the United States. A novel fusion toxin treatment, diphtheria toxin GM-CSF (DT-GMCSF) has been shown to selectively eliminate leukemic repopulating cells that are critical for the formation of AML. We previously showed that DT-GMCSF treatment of U937 cells, an AML cell line, causes activation of caspases and the induction of apoptosis.

**Methods and Findings:**

In this study we further investigate the mechanisms of cell death induced by DT-GMCSF and show that, in addition to the activation of caspase-dependent apoptosis, DT-GMCSF also kills AML cells by simultaneously activating caspase-independent necroptosis. These mechanisms depend on the ability of the targeted toxin to inhibit protein synthesis, and are not affected by the receptor that is targeted or the mechanism through which protein synthesis is blocked.

**Conclusions:**

We conclude that fusion toxin proteins may be effective for treating AML cells whether or not they are defective in apoptosis.

## Introduction

Acute myelogenous leukemia (AML) is the second most common leukemia with approximately 13,410 new cases annually in the United States and 8,990 deaths each year [1]. Current treatment for AML is the anti-metabolite cytosine arabinoside and anthracycline, which results in 65–85% of patients achieving complete clinical response [2], [3]. Unfortunately, most of these patients succumb to new tumors [2] because cytosine arabinoside does not effectively target the critical tumor progenitor cells, which allows the tumor to reappear over time. Thus, new therapies are needed to effectively target these long-term leukemic repopulating cells in order to eradicate AML. Granulocyte-Macrophage Colony-Stimulating Factor Receptors (GM-CSFR) are upregulated in many AMLs [4], and more than 70% of AMLs respond to Granulocyte-Macrophage Colony-Stimulating Factor (GM-CSF) [5]. Thus, one potential approach for more effectively treating AML would be to selectively target cells with increased levels of GM-CSFR. A novel fusion toxin treatment, diphtheria toxin GM-CSF (DT-GMCSF) has been shown to eliminate these long-term leukemic repopulating cells while sparing normal hemopoietic cells [4], [6].

Targeted toxins are fusion proteins that combine a targeting protein, such as a ligand for a specific receptor, and a toxic peptide derived from a bacterial pathogen [7], [8]. Diphtheria toxin (DT) is a bacterial pathogen that kills eukaryotic cells by inhibiting protein synthesis through the ADP-ribosylation of eEF-2 [9]. Previous studies from our groups show that a recombinant fusion protein consisting of DT-GMCSF selectively kills AML cells [10]. We previously showed that DT-GMCSF treatment of U937 cells, an AML cell line, causes activation of caspases and the induction of apoptosis [10] *via* a mechanism that involved the adaptor protein FADD, which regulates the extrinsic apoptotic pathway that is normally activated by ligand binding to death receptors (e.g. Fas/CD95 and the TRAIL receptors). However, because tumor cells often develop resistance to apoptosis e.g. by loss of caspases, upregulation of anti-apoptotic Bcl-2 family proteins or through increased expression of caspase inhibitors, we wished to test if other mechanisms of cell killing could also be induced in AML cells treated with DT-GMCSF. One potential mechanism through which this might be achieved is a novel caspase-independent cell death mechanism that utilizes components of the extrinsic apoptosis pathway to induce a programmed necrotic cell death mechanism that has been termed necroptosis [11], [12].

We show that DT-GMCSF kills AML cells by simultaneously activating both caspase dependent apoptosis and caspase-independent necroptosis. Because targeted toxins also activate the receptors that they are targeted through and these signals might regulate the death mechanisms, we also tested whether the native DT protein and the chemical protein synthesis inhibitor cycloheximide work through the same mechanisms. We conclude that fusion toxin proteins may be effective for treating AML cells whether or not they are defective in apoptosis because they can activate multiple death pathways including necroptosis as well as apoptosis. These mechanisms depend on the ability to inhibit protein synthesis, not the receptor that is targeted or the mechanism through which protein synthesis is blocked.

## Results

### DT-GMCSF Reduces Anti-Apoptotic Proteins and Activates Apoptosis

**Figure 1:**
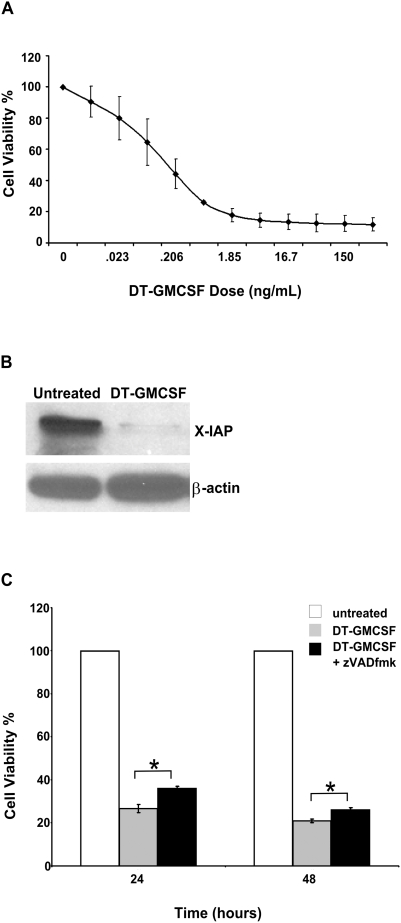
DT-GMCSF reduces anti-apoptotic proteins and activates apoptosis. (A). Cell viability of U937 cells as determined by MTS assays after 48 hours of treatment with DT-GMCSF 3-fold serial dilution (mean+/−SEM from 3 replicates). (B). Analysis of X-IAP protein levels in U937 cells in response to DT-GMCSF for 12 hours by western blotting. (C). Pre-treatment with zVADfmk (50 µM) for one hour protects U937 cells against DT-GMCSF (150 ng ml^−1^) toxicity as determined by MTS assays (mean+/−SEM from 3 replicates) (*P<0.05).

Previous studies in our laboratory indicated that DT-GMCSF can activate caspases in U937 cells [10]. Because diphtheria toxin is a potent inhibitor of protein synthesis, this may be associated with the downregulation of anti-apoptotic proteins with rapid turnover, such as, the caspase inhibitor XIAP. To confirm these conclusions and test the functional significance of the apoptosis, U937 cells were exposed to increasing doses of DT-GMCSF for 48 hours and cell survival was determined with a MTS cell viability assay (Fig. 1A). Consistent with the idea that this cell death is associated with apoptotic characteristics, chromatin was digested into a nucleosomal ladder (data not shown). Western blot analysis showed that 150 ng/ml of DT-GMCSF causes a marked decrease in XIAP levels (Fig. 1B), which supports the notion that DT-GMCSF reduces anti-apoptotic proteins and can activate caspase-dependent apoptosis. If caspase activation and apoptosis is the mechanism of DT-GMCSF-induced death, caspase inhibition should protect against the drug. However, pre-treatment with the pan-caspase inhibitor zVADfmk at 50 µM provided only partial protection against DT-GMCSF induced cell death (Fig. 1C). These data indicate that caspase-independent, non-apoptotic mechanisms also contribute to the DT-GMCSF-induced death.

### DT-GMCSF Activates Necroptosis as an Additional Death Mechanism

**Figure 2:**
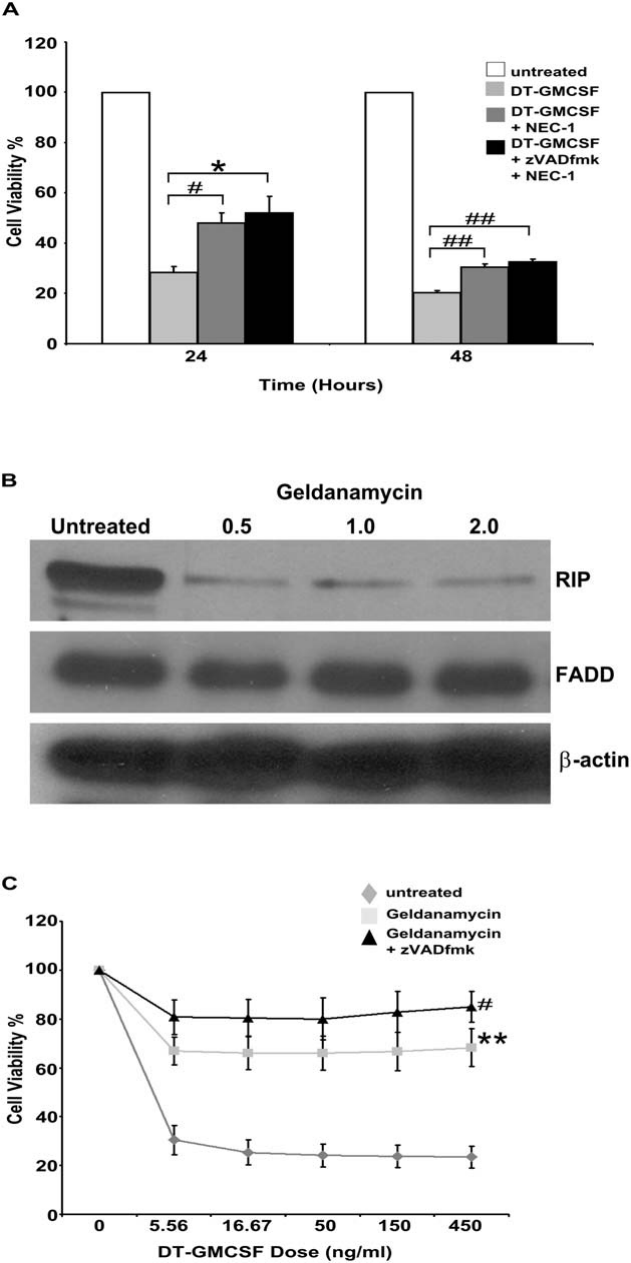
DT-GMCSF activates necroptosis as an additional death mechanism. (A). MTS assays quantifying cell viability of U937 cells in response to DT-GMCSF (150 ng ml^−1^) in the presence of Necrostation-1 (50 µM) alone or in combination with zVADfmk (50 µM) (mean+/−SEM from 4 replicates). (B). Western blotting for RIP in the presence of the HSP90 inhibitor Geldanamycin at increasing concentrations. Geldanamycin specifically depletes RIP levels while not affecting other proteins important for apoptotic and necrotic cell death such as FADD. (C). Pre-treatment with Geldanamycin (0.5 µM) either alone or in combination with zVADfmk (25 µM) protects U937 cells against DT-GMCSF induced cell death as determined by MTS assays (mean+/−SEM from 3 replicates) (* P<0.05) (**P<0.01) (#P<0.005) (##P<0.001).

Our previous data indicated that DT-GMCSF induced cell death was dependent on the adaptor protein FADD [10]. Therefore, we hypothesized that the caspase-independent death mechanism might arise through a process that is also induced by components of the extrinsic pathway. Yuan and colleagues characterized another cell death pathway activated by TNF−α when caspases were inhibited, which they termed necroptosis. This alternative cell death pathway utilizes FADD to recruit the protein kinase RIP to execute a non-caspase-dependent death signal [13], [14]. Additionally, they identified a specific inhibitor to this form of cell death called Necrostatin-1 (NEC-1) [11]. To test if necroptosis contributes to the DT-GMCSF-induced death, we pre-treated cells with the caspase inhibitor zVADfmk and NEC-1 separately and together and asked if the combination of inhibitors was more effective than either alone. DT-GMCSF induced cell death was more effectively inhibited by the combination of inhibitors (Fig. 2A) suggesting that necroptosis contributes to the caspase-independent death.

### Inhibition of RIP Protects Against DT-GMCSF

If DT-GMCSF activates necroptosis, then we should be able to recapitulate protection against DT-GMCSF seen with NEC-1 by removing RIP, which interacts with FADD [11], [13] and is required for necroptosis [11]. We therefore used geldanamycin, which inhibits HSP70-mediated RIP stabilization [15] to down-regulate RIP expression (Fig. 2B). Geldanamycin alone protected against DT-GMCSF induced cell death (Fig. 2C) and combining geldanamycin with zVADfmk provided increased protection. Inhibition of cell death was not due to a down regulation of FADD as geldanamycin did not affect FADD levels (Fig. 2B). These data suggest that NEC-1 and geldanamycin both protect against DT-GMCSF induced cell death by inhibiting necroptosis, the non apoptotic death mechanism.

### Diphtheria Toxin Alone is Sufficient to Activate Both Cell Death Pathways

**Figure 3:**
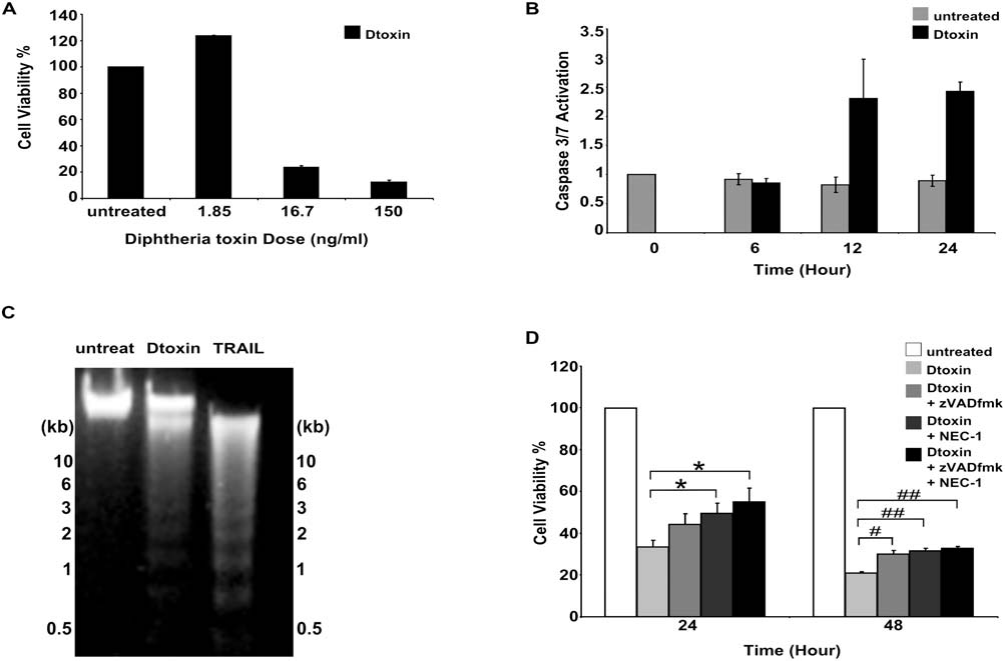
Diphtheria toxin alone is sufficient to activate both cell death pathways (mean+/−SEM from 3 replicates). (A). MTS assays measuring U937 cell viability in response to Diphtheria toxin dose curve. (mean+/−SEM from 3 replicates) (B). Diphtheria toxin (150 ng ml^−1^) activates caspases in U937 cells as determined using a caspase 3/7 specific luminogenic substrate (mean+/−SEM from 3 replicates). (C) DNA from U937 cells treated with Diphteria toxin (150 ng ml^−1^) or TRAIL (150 ng ml^−1^)/cycloheximide (0.5 µg/ml) were run on a 2% agarose gel to detect nucleosomal laddering as a marker of apoptosis. (D). Pretreating U937 cells with zVADfmk (50 µM) and/or Necrostatin-1 (50 µM) provides protection against Diphtheria toxin (150 ng ml^−1^) as measured by MTS assays (mean+/−SEM from 4 replicates). (* P<0.05) (**P<0.01) (#P<0.005) (##P<0.001).

DT-GMCSF utilizes the GMCSF receptor to target AML cells, and we previously showed that this leads to activation of GMCSF Receptor signaling {Horita, 2008 #1449}{Thorburn, 2004 #1191}, which activates various survival and proliferative pathways that can result in upregulation of anti-apoptotic proteins [17]. It is therefore possible that inhibition of the apoptotic pathway as a consequence of the receptor activation may force the tumor cells to die by activation of the alternative necroptotic pathway. To test this hypothesis, we asked if the diphtheria toxin portion of the drug is sufficient to activate both cell death pathways, or alternatively if signaling through the GMCSF receptor determines which pathways are activated. We treated U937 cells with native diphtheria toxin obtaining dose-dependent cell death similar to DT-GMCSF treatment (Fig. 3A). We detected caspase 3 and 7 activity with diphtheria toxin treatment that mimicked those seen with DT-GMCSF (Fig. 3B). We also observed distinct nucleosomal laddering as a result of chromatin digestion when we treated cells with diphtheria toxin at 150 ng/ml, which is similar to U937 cells that have been treated with the bona fide activator of the extrinsic apoptosis pathway TRAIL (Fig. 3C). These data suggest that diphtheria toxin alone is sufficient to activate the apoptosis pathway in U937 cells. However, as with DT-GMCSF, addition of NEC-1 alone or in combination with zVADfmk provided significant protection against diphtheria toxin induced cell death (Fig. 3D) indicating that the toxin activates both cell death mechanisms regardless of GMCSF receptor activation.

### Inhibition of Protein Synthesis is Sufficient to Activate Apoptosis and Necroptosis in U937 Cells

**Figure 4:**
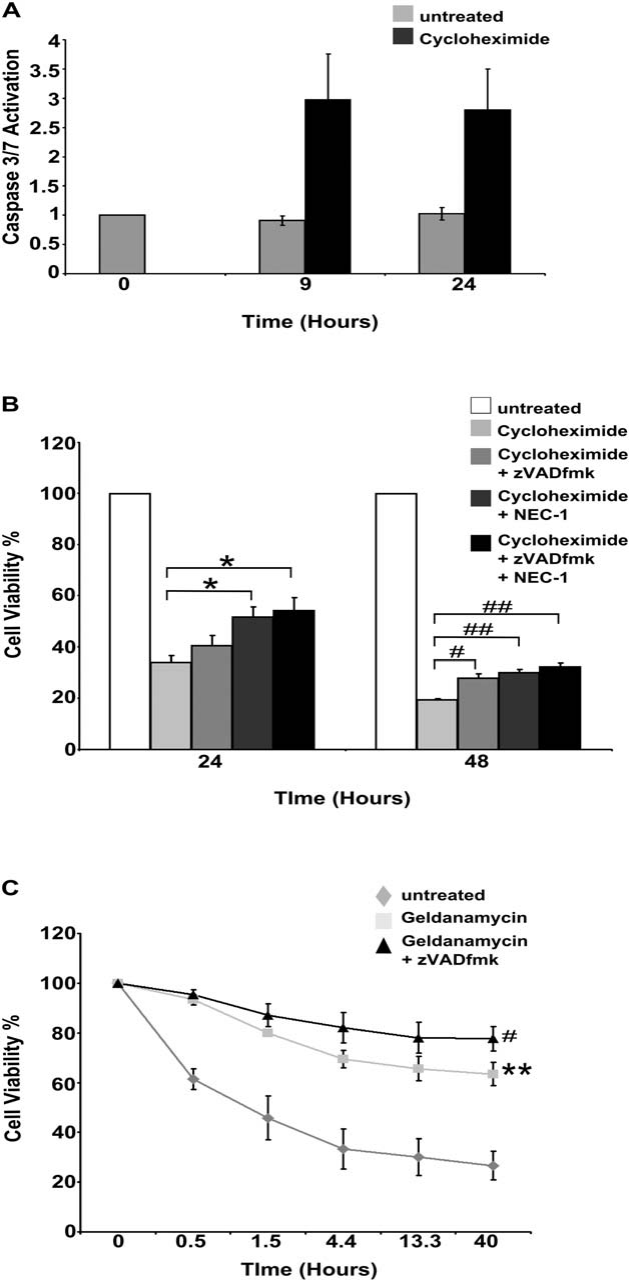
Inhibition of protein synthesis is sufficient to activate apoptosis and necroptosis in U937 cells. (A). Cycloheximide (5 µg ml^−1^) induce caspase activity in U937 cells in a manner similar to both Diphtheria toxin and DT-GMCSF as determined using a caspase 3/7 luminogenic assay (mean+/−SEM from 3 replicates). (B). Cell viability of U937 cells in response to cycloheximide (5 µg ml^−1^) in the presence of zVADfmk (50 µM) and/or Necrostatin-1 (50 µM) was measured using MTS assays (mean+/−SEM from 4 replicates). (C). MTS assays were used to measure the ability of Geldanamycin (0.5 µM) and zVADfmk (25 µM) to protect against cycloheximide (5 µg ml^−1^) induced cell death in U937 cells (mean+/−SEM from 3 replicates) (* P<0.05) (**P<0.01) (#P<0.005) (##P<0.001).

Diphtheria toxin ADP-ribosylates eEF-2 to shut down protein synthesis and an ADP-ribosylation inhibitor completely blocked targeted toxin induced cell death (data not shown). We therefore asked if another mechanism of protein synthesis inhibition is sufficient to activate apoptosis and necroptosis by treating cells with high levels (5 µg/ml) of cycloheximide (a chemical inhibitor of protein synthesis that works by interfering with the 80S ribosome), which as with DT-GMCSF significantly decreased XIAP levels in U937cells (data not shown). Cycloheximide induced effector caspase activity similar to that observed with DT-GMCSF and diphtheria toxin treatment (Fig. 4A). These data indicate that inhibition of protein synthesis is sufficient to induce caspase-dependent apoptosis in U937 cells. Moreover, similar to DT-GMCSF, pre-treatment with zVADfmk alone was not able to provide complete protection against cell death (Fig. 4B), while addition of NEC-1 (Fig. 4B) or RIP depletion with geldanomycin (Fig. 4C) provided additional protection. These data suggest that any mechanism of inhibiting protein synthesis activates both apoptosis and necroptosis in these cells.

### Necrostatin Protects Against DT-GMCSF in Other AML cell Lines

**Figure 5:**
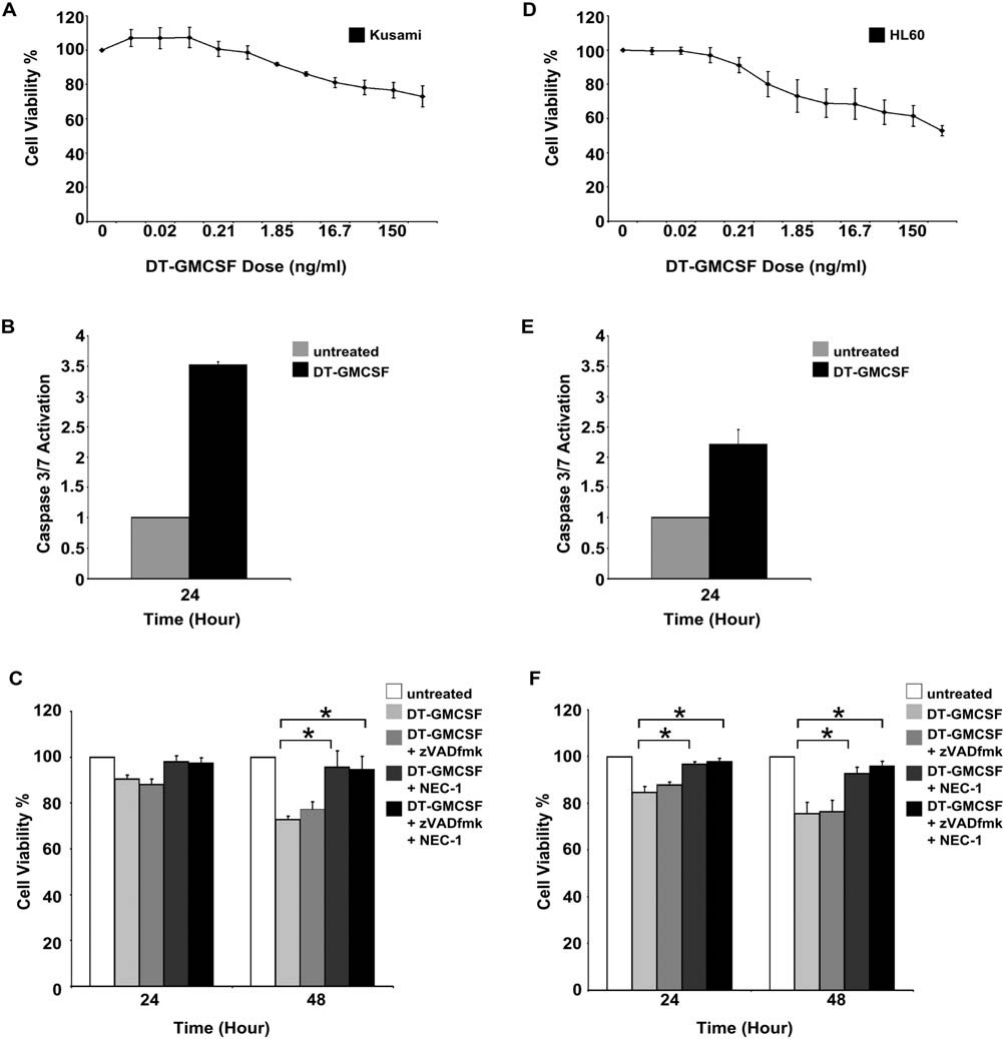
DT-GMCSF kills other AML cells by necroptosis. (A). Cell viability of Kusami cells as determined by MTS assays after 48 hours of treatment with DT-GMCSF 3-fold serial dilution (mean+/−SEM from 3 replicates). (B). DT-GMCSF (450 µg ml^−1^) induce caspase activity in Kusami cells as determined using a caspase 3/7 luminogenic assay (mean+/−SEM from 3 replicates). (C). Cell viability of Kusami cells in response to DT-GMCSF (450 µg ml^−1^) in the presence of zVADfmk (50 µM) and/or Necrostatin-1 (50 µM) was measured using MTS assays (mean+/−SEM from 3 replicates). (D). Cell viability of HL60 cells as determined by MTS assays after 48 hours of treatment with DT-GMCSF 3-fold serial dilution (mean+/−SEM from 3 replicates). (E). DT-GMCSF (450 µg ml^−1^) induce caspase activity in HL60 cells as determined using a caspase 3/7 luminogenic assay (mean+/−SEM from 3 replicates). (F). Cell viability of HL60 cells in response to DT-GMCSF (450 µg ml^−1^) in the presence of zVADfmk (50 µM) and/or Necrostatin-1 (50 µM) was measured using MTS assays (mean+/−SEM from 3 replicates). (* P<0.05).

We next asked whether DT-GMCSF's ability to activate necroptosis occurs in other AML cell types. Treatment of Kusami cells (Fig. 5A) and HL60 cells (Fig. 5D) with DT-GMCSF showed that the drug also killed these AML cell lines (although to a lesser extent than the U937 cells). We then measured caspase activity in both cell lines treated with DT-GMCSF and found similar activation to that measured in U937 cells (Fig. 5B,E). Finally, we treated cells with DT-GMCSF in the presence of zVADfmk and NEC-1. Interestingly, both cell types had increased viability in the presence of NEC-1, however, there was little protection with zVADfmk (Fig. 5C,F). These data suggest that the necroptosis pathway is activated along with the apoptosis pathway in other AML cell lines by DT-GMCSF and emphasize that inhibition of caspase activity doesn't necessarily lead to protection.

## Discussion

As with other anti cancer agents, better understanding of how targeted toxins kill tumor cells may provide valuable insights to optimize their clinical use. Previous data from our laboratory [10] and others (reviewed in [18]) showed that, like many other anti-cancer agents, targeted fusion toxin proteins often kill tumor cells by activating caspase dependent apoptosis. In this study we extend these findings to show that in addition to caspase-dependent apoptosis, a targeted or native DT protein and a chemical inhibitor of protein synthesis also kill AML cells by activating necroptosis, which is a non-apoptotic programmed cell death mechanism that requires the protein kinase RIP. In U937 cells, both mechanisms contribute similarly to cell death because selective inhibition of necroptosis with NEC-1 or depletion of RIP provided some protection, which was increased by addition of a caspase inhibitor. However, in other cell types the protection by necrostatin was greater than that provided by the caspase inhibitor. The mechanism of action of DT-GMCSF is dependent on the diphtheria toxin and inhibition of protein synthesis alone, as both native DT and cycloheximide were sufficient to activate both cell death mechanisms similarly to DT-GMCSF. This leaves us with the question of how protein synthesis inhibition activates both apoptotic and non-apoptotic cell death mechanisms. For apoptosis, depletion of proteins with rapid turn-over that directly inhibit the apoptosis machinery, such as, XIAP provides a plausible explanation and we suggest that the necroptosis machinery may also be held in check by inhibitors that are depleted in cells where protein synthesis is blocked.

Although DT-GMCSF can kill AML cells in both an apoptotic and necroptotic fashion, blocking these two pathways may only delay the death as shown by lesser protection at later time points (Figure 2A). Additionally, we previously found that certain cell lines that don't activate caspases at all in response to targeted DT may die by a slow cell death mechanism that does not involve the release of cytosolic proteins that occurs in necroptosis [16], [19]. Together, these findings indicate that despite the fact that targeted DT fusion toxins work by inhibiting a fundamental cell activity (protein synthesis), multiple death mechanisms can be activated that may be more or less important depending upon the particular tumor cell that is being targeted. Thus, although our data clearly show that many tumor cells die in response to targeted toxins by activating efficient and rapid death pathways such as necroptosis or apoptosis, the drugs may be effective even if these pathways are blocked. In fact, we have found that the most effective ways to block targeted DT proteins from killing tumor cells effectively enough, so that tumor cells can regain the capacity for clonogenic growth after removal of the toxin, is by preventing drug uptake by depleting target signaling receptor [19] or by inhibiting the catalytic subunit of diphtheria toxin using an ADP-ribosylation inhibitor (unpublished data). These data suggest that unlike with some other tumor-selective agents such as TRAIL receptor-targeted drugs, where true resistance can be achieved by inhibiting apoptosis [20], it may be more difficult for tumor cells to evolve a mechanism of avoiding targeted toxin-induced death.

DT-GMCSF has shown clinical efficacy when used to treat patients with AML, however, it also caused liver toxicity due to GMCSF receptor expression in monocytes and macrophages [21]–[23]. It may be possible to avoid the toxicity problems using a modified version of DT-GMCSF (DTU2GMCSF) that provides dual targeting specificity to AML cells by replacing the furin cleavage site within the toxin with a urokinase plaminogen activator (uPA) cleavage site [22], [23]. Other fusion toxin drugs utilizing diphtheria toxin, e.g. DT-IL3, are also being tested in clinical trials to treat AML [24]. Our data show that the diphtheria toxin subunit is responsible for the type of cell death induced in AML cells, thus these other drugs that incorporate diphtheria toxin should work by a similar mechanism to DT-GMCSF. Therefore, we anticipate that the multiple death mechanisms that we have observed will be induced by these agents and that our arguments about the difficulty of evolving true resistance to these drugs will also apply to these different targeted toxins.

In summary, we show that at least two programmed cell death mechanisms (apoptosis and necroptosis) are activated by GMCSF-targeted diphtheria toxin in AML cells. Consistent with previous studies using other toxins in different cell types, the particular pathway that is activated depends, to at least some extent, on the cell line that is being tested, however the most important finding is that blocking any one death mechanism is not sufficient to prevent tumor cell killing, and instead just causes the cell to die from the other mechanism or by slower death pathway. In the clinical context we propose that these attributes will make it difficult for tumor cells to evolve resistance mechanisms other than down-regulation of the targeted receptor. Since the receptors that are targeted with this approach are usually chosen because they are important for driving tumor growth, one would expect selective pressure against downregulation of the receptor, which should increase the efficacy of the treatment if a useful therapeutic window can be developed by avoiding targeting normal tissues. Therefore, our data also emphasize that the practical application of targeted toxins in the clinic will require that we are successful in our efforts to make the tumor targeting as selective as possible and/or using delivery methods that are able to avoid susceptible normal tissues.

## Materials and Methods

### 

#### Reagents

Human U937 cells, Kusami and HL60 cells were obtained from American Type Culture collection and maintained in RPMI 1640 growth medium (HyClone, Logan, UT) supplemented with 10% fetal bovine serum (FBS) (HyClone, Logan, UT) in a 5% CO_2_ humidified atmosphere at 37°C. DT-GMCSF was prepared as described previously [13]. Unless otherwise noted, chemicals and reagents were obtained from Sigma Chemical Co. (St. Louis, MO). Cycloheximide at 5 µg/ml, geldanamycin (Alexis Biochemicals, San Diego, CA) at 0.5 µM, 1 µM, or 2 µM, zVADfmk (Alexis Biochemicals, San Diego, CA) at 25 µM or 50 µM, Necrostatin-1 (Biomol International, Plymouth Meeting, PA) at 50 µM, diphtheria toxin (Calbiochem, La Jolla, CA), TRAIL (R&D Systems, Inc., Minneapolis, MN) at 150 ng/ml.

#### Immunoblotting

At the indicated times, 1×10^6^ cells were harvested, and lysates were prepared by boiling in SDS buffer 10 minutes prior to gel electrophoresis. Lysates were resolved on 12% SDS-polyacrylamide gels. Following electrophoresis, proteins were transferred to nitrocellulose membrane. Blots were incubated with antibodies that recognize X-IAP (Cell Signaling Technologies, Danvers, MA), RIP (BD Pharmigen), and FADD (BD Pharmigen ), and β-Actin (Sigma, St Louis, MO). Blots were then incubated with anti-rabbit or anti-mouse horseradish peroxidase-conjugated secondary antibodies (Cell Signaling Technologies, Danvers, MA). Detection was performed using the chemiluminescent ECL reagent (Millipore Corporation, Billerica, MA), and developed on Blue Basic Autorad Film (ISC Bioexpress, Kaysville, UT).

#### DNA Laddering

At the indicated times, 1×10^6^ cells were pelleted at 900 g for 5 minutes. The supernatant was discarded and pellets were washed with PBS. Cells were then resuspended in SDS lysis buffer (add fresh 0.5 mg/ml proteinase K) and incubated at 55°C for one hour. Cell debris were pelleted and removed. 5 µl of RNase A were then added (1 mg/ml) to the supernatant and incubated at 55°C for one hour. The samples were next incubated at 80°C for 20 minutes to inactivate the proteinase K. 15 µL of sample were loaded on a 2% agarose gel (0.5 µg/ml ethidium bromide) and visualized with UV transillumination on a Molecular Imager Gel Doc XR system (Bio-Rad Laboratories, Hercules, CA).

#### Caspase 3/7 Activity

Cells were plated in a 96 well black-walled plate at a concentration of 30,000 cells per well. Drugs were added in triplicate at timepoints of interest. One hour prior to the end of assay Caspase-Glo 3/7 reagent (Promega, Madison, WI) was added to each well at a 1∶1 ratio according to manufacturers' instructions. Cells were then incubated in the dark for one hour. Samples were measured using a Veritas microplate luminometer (Turner BioSystems, Sunnyvale, CA).

#### MTS cell viability

Cells were plated in a 96 well format at 30,000 cells per well and treated with the indicated drugs in triplicate wells. One hour prior to the end of the experiment cells were treated with 20 µl of 3-(4,5-dimethylthiazol-2-yl)-5-(3-carboxymethoxyphenyl)-2-(4-sulfophenyl)-2H-tetrazolium, inner salt (MTS) (Promega, Madison, WI) according to manufacturers' instructions and measured with a Bio-Rad Benchmark Plus Microplate Spectrophotometer (Bio-Rad Laboratories, Hercules, CA) at an absorbance value of 490 nm. For samples where inhibitors (NEC-1, geldanamycin etc.) were used, normalization to control cells treated with the inhibitor alone was carried out to account for any toxicity caused by the inhibitor itself.
